# Integrated multisectoral interventions to mitigate the risk of low birth weight in low- and middle-income country settings: Implementation considerations for programs from a WHO expert consultation

**DOI:** 10.7189/jogh.14.03033

**Published:** 2024-09-13

**Authors:** WHO Consultative Group on multisectoral programmatic interventions to reduce the risk of low birth weight, Shabina Ariff, Per Ashorn, Ulla Ashorn, Rajiv Bahl, Hana Bekele, Yemane Berhane, Nita Bhandari, Zulfiqar Bhutta, Ranadip Chowdhury, Parul Christian, Gary L Darmstadt, Ayesha De Costa, KE Dickson, Christopher Duggan, Wafaie W Fawzi, G Justus Hofmeyr, Caroline Homer, Patricia Hunter, Pui-Ying Iroh Tam, Yunhee Kang, Joanne Katz, Annariina Koivu, Anne CC Lee, Jose Carlos Martines, Allisyn Moran, Yvonne Muthiani, Helga Naburi, Pieta Näsänen-Gilmore, Uttara Partap, Usha Ramakrishnan, Joao Paulo Souza, Sunita Taneja, Marleen Temmerman, Dilys Walker, Eleonor Zavala

## THE PERSISTENT PROBLEM OF LOW BIRTH WEIGHT

In 2020, 19.8 million newborns, an estimated 14.7% of all births globally that year, were born with a low birth weight (LBW) (<2500 g) [1]. LBW is associated with an increased risk of mortality and accounts for about half of all neonatal deaths [2]. An estimated 91% of all LBW neonates are born occur in low- and middle-income country settings, most commonly in South Asia and sub-Saharan Africa [3]. LBW newborns who survive the neonatal period are at an increased risk of developmental impairment in infancy and childhood [4–6], and cardiometabolic disease [7] in later life. Despite one in seven newborns being born LBW and exposed to the risks described above, there has been very little progress globally in reducing the prevalence of LBW over the last 20 years, with an average annual reduction rate of only 0.3% per year [1]. The World Health Assembly has set a nutrition target to reduce LBW by 30% between 2012–30.

**Figure 1:**
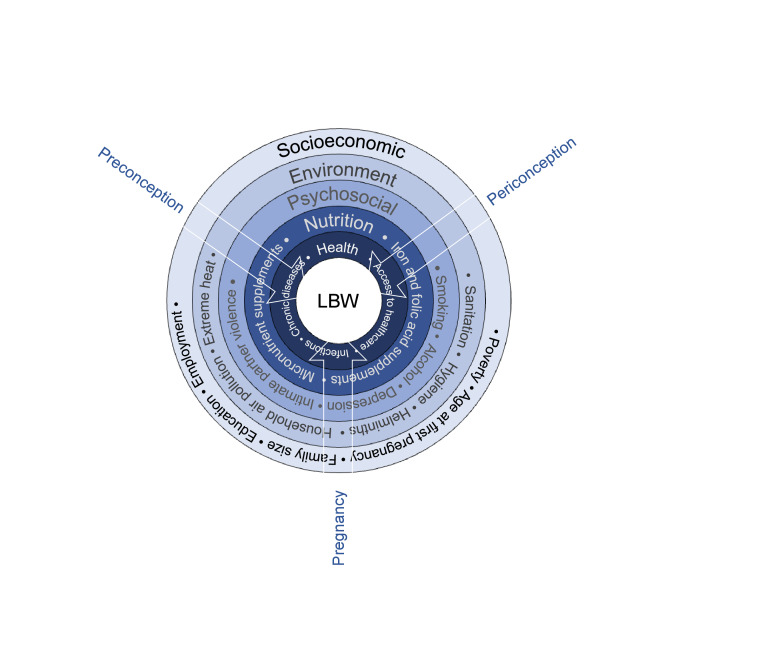
Conceptual framework of risk factors for low birth weight.

The focus so far has been on single interventions aimed at reducing the risk of LBW, which have had varying effectiveness [8]. More recently, evidence has emerged on the combined impact of multisectoral interventions to reduce the risk of LBW and subsequently improve neurodevelopmental outcomes (**Table 1**) [9,13,14]. There is also an increased understanding of the possible pathways resulting in LBW and its sequelae, including health, nutrition, psychosocial, environmental, and socioeconomic factors (**Figure 1**), and studies incorporating multiple interventions are under way [10,15]. In light of these initiatives, World Health Organization (WHO) convened a global consultation on this topic in December 2021. This meeting brought together stakeholders from academia, non-governmental organisations, and global partners, including the World Bank, United Nations Children’s Fund (UNICEF), national nutrition institutes, ministries of health and donors from different countries.

**Table 1:** Multisectoral interventions used in recent studies to reduce the risk of low birth weight

Study and location	Health		Psychosocial	Nutrition		Environment		Time periods	
WINGS, India [9]	At least eight antenatal care contacts in pregnancy screen and treat GHT or preeclampsia, GDM, hypothyroidism, UTI, RTI, calcium supplement		Promote positive thinking and problem-solving skills	Iron, folic acid, and multiple micronutrients, protein-energy supplements to undernourished women during preconception and to meet additional requirements during pregnancy		Promote personal, menstrual, and hand hygiene		Preconception, pregnancy	
ENAT-ACIPH – Ethiopia (Amhara) [10]	Urinary tract infection, gonorrhoea-chlamydia, BV-trichomonas screening and treatment; presumptive deworming, stool screening and treatment		NA	Nutrition counselling, iron-folic acid, routine supply of iodised salt, micronutrient fortified balanced energy protein supplement to undernourished women		NA		Pregnancy	
ENAT – Ethiopia (Amhara-Oromia) [11]	Clinical skills strengthening training, point-of-care testing training, availing essential equipment and supplies, deworming		Mentoring and supportive supervision	Screening pregnant mothers for anaemia and malnutrition, iron and folic acid supplements		NA		Pregnancy	
Bolsa Família Program, Brazil [12]	Expanded conditional cash transfer program	Expanded conditional cash transfer program		Expanded conditional cash transfer program	NA		Preconception, pregnancy	

ACIPH – Addis Continental Institute of Public Health, BV – bacterial vaginosis, ENAT – Enhancing Nutrition and Antenatal Infection Treatment for Maternal and Child Health, GDM – gestational diabetes, GHT – gestational hypertension, NA – not available, RTI – reproductive tract infection, UTI – urinary tract infection

The aims of this WHO meeting were to discuss programmatic considerations related to the integrated implementation of effective interventions across different sectors (nutrition, health, psychosocial, environment, socioeconomic) to reduce the risk of LBW and highlight knowledge gaps and future research priorities, to support planniny by governments on investments in multisectoral prevention programs. In this paper, we describe the output from the consultation as a framework to support program managers/health departments intending to initiate a multisectoral program to reduce the risk of LBW.

### The emerging evidence on multisectoral interventions and low birth weight

A recent study to specifically assess the impact of multisectoral interventions – multiple domains incorporated into a bundled intervention – in the preconception and pregnancy periods on LBW outcomes was the Women and Infants Integrated Interventions for Growth Study (WINGS) in India [9]. WINGS was an individually randomised factorial trial that incorporated a package of interventions that included health (screening and treatment of maternal medical conditions, ≥8 antenatal care contacts in pregnancy), nutrition (iron, folic acid, multiple micronutrients, and protein-energy supplements to undernourished women during preconception, and to meet the additional requirement during pregnancy), psychosocial care, and environment (personal, menstrual, and hand hygiene) [9]. In WINGS, receipt of preconception interventions were associated with a significantly lower risk of LBW compared to groups without (incidence rate ratio (IRR) = 0.85, 98.3% confidence interval (CI) = 0.75, 0.97; absolute risk reduction (ARR) = –3.80, 98.3% CI = –6.99, –0.60). Interventions implemented across several domains during preconception and pregnancy reduced the risk of LBW by 24% (IRR = 0.76, 98.3% CI = 0.61, 0.97; ARR = −5.59, 98.3% CI = –10.32, –0.85), which was twice as great as the reduction seen in prior intervention trials that focused only on a single domain, nutrition [16,17]. In addition, children whose mothers received multisectoral interventions in the preconception and pregnancy periods had higher cognitive (mean difference (MD) = 2.60; 98.3% CI = 1.08, 4.12), language (MD = 3.46; 98.3% CI = 1.65, 5.27), motor (MD = 2.31; 98.3% CI = 0.93, 3.69), and socioemotional (MD = 5.55; 98.3% CI = 2.66, 8.43) scores at 24 months than did those in the control group [14]. In Ethiopia (Amhara-Oromia), a cluster randomised trial of a system-strengthening intervention focused on training, availability of commodities and supportive supervision of staff to deliver health and nutrition interventions to pregnant mothers demonstrated a significantly higher mean birth weight in the intervention compared to the control arm (MD = 108.00; 95% CI = 91.30, 124.60) [11]. In rural Sierra Leone, a randomised, controlled clinical effectiveness trial of a ready-to-use supplementary food plus anti-infective therapies compared to standard therapy in undernourished pregnant women led to higher birth weight in newborns among women receiving the intervention compared to standard of care (MD = 70.00; 95% CI = 0.02, 0.12), as well as greater weight gain in women (MD = 40.00; 95% CI = 9.70, 71.0) [18]. Another multisectoral intervention, Enhancing Nutrition and Antenatal Infection Treatment for Maternal and Child Health in Amhara, Ethiopia delivered packages of interventions to optimise nutrition (counselling, iron folic acid, iodised salt and micronutrient balanced energy protein supplementation to undernourished women) and maternal infection management (genitourinary tract infection screening-treatment, deworming) through the existing Ethiopian health system [10]; results are forthcoming.

Additional data comes from the Bolsa Família Program [12], a conditional cash transfer program in Brazil where low-income participating families receive monthly payments if children are enrolled at school and vaccinated, and women and children attend scheduled health visits, including antenatal, postnatal and routine childcare follow-up [19]. A significant co-intervention in the Brazilian context is the Family Health Strategy, a family and community health approach for primary health care within the public Unified Health System that functions as a delivery platform for intersectoral intervention [19]. Using national data sets to evaluate the correlation between an expanded conditional cash transfer program for maternal and newborn health, researchers found that children born into cash transfer recipient households were less likely to have LBW (odds ratio (OR) = 0.93; 95% CI = 0.92, 0.94) and very LBW (OR = 0.87; 95% CI = 0.84, 0.89) [19]. Maternal benefits included a positive correlation between conditional cash transfers and improved prenatal care [19]. Given that studies done in Brazil have previously demonstrated a link between LBW and socioeconomic conditions, low maternal education, being a single mother, and being <20 or >34 years of age, it is likely that the cash transfer under the Bolsa Família Program mitigated risks across multiple domains.

Although intervention delivery in trials tends to be more intensive than in programs, this emerging evidence from both trials and programs suggests that the risk of LBW can be substantially reduced through multisectoral programmatic interventions focused on women of reproductive age [20], especially in the preconception and pregnancy periods. Although it is unclear which interventions explain specific proportions of the impact, the results offer an approach to reduce LBW in newborns.

### Programmatic planning of multisectoral interventions to reduce the risk of low birth weight – deliberations from the global consultation

**Figure 2:**
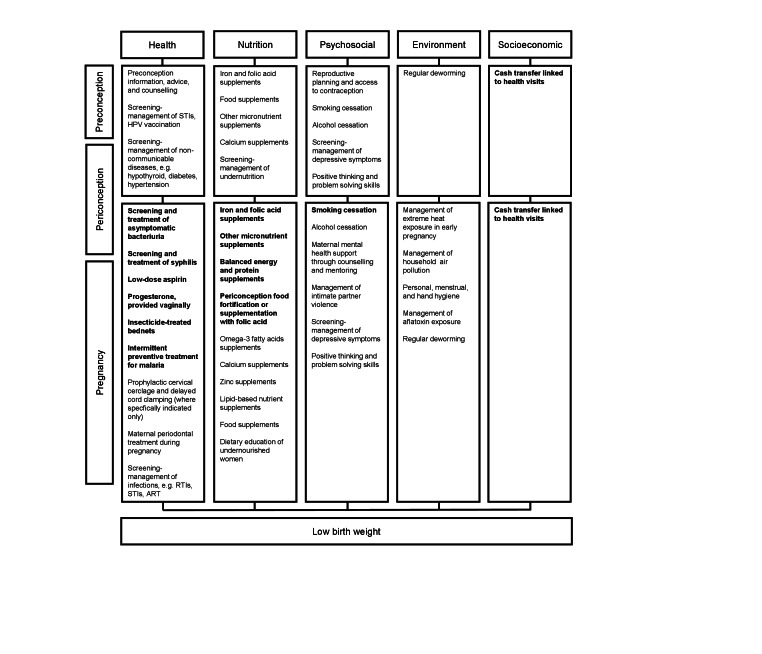
Domains for interventions to reduce the risk of low birth weight – a framework. Only interventions listed in bold have proven effect [8,19,20]. This list of potential interventions is not comprehensive. ART – antiretroviral therapy, HPV – human papillomavirus, RTIs – reproductive tract infections, STIs – sexually transmitted infections.

The design of a multisectoral program should consider the burden of risk factors contributing to LBW (e.g. maternal ill health, education and employment, adolescent pregnancy, nutritional deficiencies, infections, poor access to health care, environmental challenges and others) within a particular context (**Figure 2**). Attention to these contextual factors will facilitate the selection of the optimal balance of interventions that are both appropriate to, feasible for, and acceptable in the setting. Careful consideration needs to be given to the choice of target population and life stage, planning/utilising the most appropriate platforms for program delivery pertinent to each group, ensuring the composition and quality of the package of interventions and its high coverage and adherence, considering the human resources required, taking into consideration the choices of women, costs, measurable outcomes, and ensuring a strong level of multisectoral coordination at all levels. Key considerations need to be given to health system capacity, which includes human resources, financing, service delivery, health information systems, access to essential medicines, and leadership/governance.

### National-level planning for multisectoral programs addressing low birth weight

When planning for multisectoral, the interventions to be included in a package to address low birth weight, one should consider the risk factors for LBW and the population-attributable risk for each of the factors in a setting [21].

It is important for policymakers and program managers in ministries of health in settings with a significant burden of LBW to consider strategies for coordination when planning to implement multisectoral interventions to reduce the risk of being born LBW. The period of life for women-focused interventions to improve LBW outcomes extends from preconception (before pregnancy occurs) through pregnancy, allowing for a continuum-of-care approach of integrated service delivery linking the health and well-being of mothers and infants [22]. Adolescents also need to be considered, as they are mothers to 21 million newborns a year (>10% of all births) [23], and a young maternal age of ≤16 years is a strong predictor for LBW (OR = 1.96; 95% CI = 1.35, 2.83) [24].

While it may be more efficient to reach intended beneficiaries through a single delivery platform, this may not be possible with more diverse interventions. A variety of delivery approaches may need to be considered, depending on the interventions selected, the target group [25] and population risk [21]. Delivery of the package can be done on multiple platforms across different sectors: within the education system using school health and reproductive health education programs; within the health system through facility-based clinical care with primary-level health workers, pre-marital counselling and screening, antenatal care, and expanded intrapartum and postnatal care; or within the community through community mobilisation and engagement including community support groups, mass media campaigns, promotion of care-seeking for illness, workplace programs or referrals, and support groups for at-risk individuals [22,26].

The choice and number of delivery platforms will be driven by the needs of the different interventions incorporated into the program: platforms could be community- or facility-based, have varied involvement of the public and private sectors and should consider women’s preferences. Integrating intervention packages into existing local health programs/health care systems and other easily accessible delivery platforms is vital in overcoming barriers to service delivery and accessibility [22].

Further implementation research to address questions around planning and executing a multisectoral program can generate valuable information to inform decision-making as more countries prepare to plan and implement such programs at scale. These include defining appropriate platforms to target pre/periconception interventions, the choice of domain interventions, the approach to delivery, and the outputs and outcomes to be monitored and measured. A list of implementation research areas was identified at the consultation (**Box 1**). A cost-benefit analysis of an integrated package of interventions to reduce LBW, including preconception and pregnancy periods, demonstrates positive returns on investment (Choudhary et al., unpublished observation).

Box 1 **Implementation research: key questions relevant to multisectoral intervention program to reduce the risk of low birth weight.**

Planning considerations:
  1. What would be an appropriate time to target an intervention? How should the preconception period be defined in a particular setting if interventions are intended to also target the period before pregnancy? How would women/ adolescents in this period be identified to better target the interventions?
  2. What would be other appropriate outcomes to measure besides newborn outcomes beyond LBW, such as small-for-gestational-age, preterm birth, and stillbirth? Will ultrasound assessment for gestational age be feasible in local contexts?
  3. How could other family members, especially male partners, be encouraged to facilitate the target beneficiary’s uptake of the package on interventions?
  4. How can women’s choice of interventions and where she receives these from, be taken in consideration, e.g. private sector? Which interventions would best suit private sector engagement in a setting, if such engagement is deemed necessary?
Direct specific considerations relevant to the nutritional component of a multisectoral intervention project/program:
  1. What infrastructure needs to be in place when nutritional interventions are implemented and monitored at scale?
  2. What are strategies to ensure that the supplements provided are consumed by the intended beneficiary? Potential strategies for testing include a) packages with a picture of a pregnant woman, b) distribution on platforms where pregnant women congregate (observed consumption), and c) use of digital technologies.
  3. What would be considered when deciding on the optimal mix of (a) cooked food, (b) raw ingredients, and (c) micronutrient supplements within a project/program?
  4. How best could other sectors working with nutritional supplementation be harnessed to participate in the project/ program (e.g. agriculture extension services and homestead gardening, school meal programs, and local businesses dealing with food).
  5. How could the counselling content be developed and delivered to potential beneficiaries and their families?
Implementation considerations:
  1. When planning to implement multisectoral interventions, what parameters should a cross-departmental program lead consider when prioritising the interventions to be included?
  2. How can nutrition interventions be bundled within the antenatal care platform?
  3. What strategies to coordinate efforts are required to facilitate multisectoral interventions that form part of the bundle?
  4. How can school-based interventions be developed that retain girls in school, limit adolescent pregnancy, and improve nutrition that may be critical for preconception or for later in life at the time of pregnancy?
  5. What are the considerations when choosing the beneficiary group for programmatic implementation of interventions to prevent LBW newborns? What are ways in which the intended target group can be identified in a context?
  6. What are suitable platforms through which multisectoral interventions to prevent LBW newborns can be delivered within a program setting so that these are received by the intended beneficiary with as little burden as possible? What would be the human resources necessary?
  7. How can we measure or assess the secondary impacts of these intervention bundles on women and families?
  8. How can the receipt of multiple interventions and outcomes be effectively monitored during implementation at scale?
  9. How can local government (different sectors), local institutions and community groups be effectively engaged in a multisectoral intervention program to prevent LBW?
  10. How can the measurement of key outcomes related to the prevention of LBW within programs be improved, e.g. gestational age and birth weight?
11)Do conditional cash transfers tied to compliance with interventions to reduce LBW improve birth outcomes?
  1. How can individual interventions that have shown strong benefits or effects be implemented at scale?
  2. What are innovative ways to have a platform/s for the delivery of coordinated multisectoral interventions within a program to reduce the risk of LBW in the population?
  3. What are the ways of sustainably financing multisectoral interventions and dealing with the logistics of procurement and supply within a multisectoral program to reduce LBW in the population?

## CONCLUSION

A multisectoral approach to tackling the unchanging proportions of LBW is an important but underexplored area of implementation research and programmatic implementation. Emerging evidence indicates that multisectoral interventions across health, nutrition, psychosocial, environment, and socioeconomic domains can be delivered to women in the preconception and pregnancy periods, with demonstrated impact on reducing the risk of infants being born with LBW, improvement in maternal health outcomes, and positive returns on investment. While these interventions focus on specific health, nutrition, and broader areas like cash transfers, they need to be considered in an overall context of empowering women, including increasing agency, decision-making power, education, and access to contraceptives. It is important to note that the intervention selection varied by setting and that all available interventions come from a research context. More evidence is needed when interventions are implemented in routine programs at scale. Policymakers and program managers are encouraged to follow emerging evidence and carefully plan when considering a multisectoral program, including the appropriate domains (health, nutrition, psychosocial, environment, socioeconomic, etc.), the magnitude of the problem that a component intervention will address, cost of the interventions, ease of implementation, delivery platforms, ease of access, human resources required, and preferences of the women for how to receive services and commodities. Given the slow reduction in LBW globally and the recent synergy demonstrated with multiple interventions implemented together – health, nutrition, psychosocial, environment, and socioeconomic – on LBW reduction [9], a government focus and investment in multisectoral intervention is increasingly important in programs to achieve the World Health Assembly nutrition target to reduce LBW by 30% between 2012–30.
